# Supplementary material for: CD163 and pAPN double-knockout pigs are resistant to PRRSV and TGEV and exhibit decreased susceptibility to PDCoV while maintaining normal production performance
Source: eLife. 2020 Sep 2;9:e57132. doi: 10.7554/eLife.57132 (PMC7467724; doi:10.7554/eLife.57132)
Supplement: Supplementary file 1. [file elife-57132-supp1.docx]

**Supplementary file 1. Gen****otypes of cell lines used in somatic cell nuclear transfer**

| Cell lines | Gender | Genotype | Indels |
| --- | --- | --- | --- |
| 18# (cell colony) | ♂ | *CD163*: CCCACAGGAAACCCAGGCTGG‑‑‑‑‑‑‑‑GACATTCCCTGCTCT  *pAPN*: GCCCTGGGCATCCTGGGCATCCTCCTCGGCGTGGCGGCCGTGGCCAC  GCCCTGGGCATCCTGGGCATCCTCCTCGGC--------CGTGGCCAC | *CD163*: -8 bp/-8 bp  *pAPN*: WT/-8 bp |
| 25# (cell colony) | ♂ | *CD163*: CCCACAGGAAACCCAGGCTGG‑‑‑‑‑‑‑‑GACATTCCCTGCTCT  *pAPN*: GCCCTGGGCATCCTGGGCATCCTCCTCGGC-----GGCCGTGGCCAC  GCCCTGGGC--------------------------GGCCGTGGCCAC | *CD163*: -8 bp/-8 bp  *pAPN*: -5 bp/-26 bp |
| 89# (cell colony) | ♂ | *CD163*: CCCACAGGAAACCCAGG‑‑‑‑‑‑‑‑-‑‑‑‑//‑‑‑‑‑‑‑‑‑‑‑  *pAPN*: GCCCTGGGCATCCTGGGCATCCTCCTCGGCGTGGCGGCCGTGGCCAC  GCCCTGGGCATCCTGGGCATCCTCCTCGGCG-----GCCGTGGCCAC | *CD163*: -26 bp/-26 bp  *pAPN*: WT/-5 bp |
| 112# (cell colony) | ♂ | *CD163*: CCCACAGGAAACCCAGGCTGGTT**T**GGAGGGGACATTCCCTGCTCT  *pAPN*: GCATCCTGGGCATCCTCCTCGGCGTGGC---------CACCATCATC  GCATCCTGGGCATCCTCCTCG---------------------**C**CATC | *CD163*: +1 bp/+1 bp  *pAPN*: -9 bp/-22 bp,+1 bp |
| 1145# (ear fibroblasts) | ♂ | *CD163*: CCCACAGGAAACCCAGGCTGG‑‑‑‑‑‑‑‑GACATTCCCTGCTCT  *pAPN*: GCCCTGGGCATCCTGGGCATCCTCCTCGGC-----GGCCGTGGCCAC  GCCCTGGGC--------------------------GGCCGTGGCCAC | *CD163*: -8 bp/-8 bp  *pAPN*: -5 bp/-26 bp |
