# Supplementary material for: CD163 and pAPN double-knockout pigs are resistant to PRRSV and TGEV and exhibit decreased susceptibility to PDCoV while maintaining normal production performance
Source: eLife. 2020 Sep 2;9:e57132. doi: 10.7554/eLife.57132 (PMC7467724; doi:10.7554/eLife.57132)
Supplement: Supplementary file 2. [file elife-57132-supp2.docx]

**Supplementary file 2. Embryo transfer data for *CD163* and *pAPN* DKO pigs**

| Recipient | Date of surgery | No. of transferred reconstructed embryos | Pregnant sows | Date of birth |
| --- | --- | --- | --- | --- |
| 118 | **12/28/2018** | **250** | **—** |  |
| 121 | **12/28/2018** | **250** | **Delivered** | **4/24/2019** |
| 119 | **12/28/2018** | **280** | **—** |  |
| 120 | **12/28/2018** | **250** | **—** |  |
| 18418 | **12/28/2018** | **250** | **—** |  |
| 18317 | **1/4/2019** | **250** | **Delivered** | **4/28/2019** |
| 123 | **1/4/2019** | **261** | **Delivered** | **4/30/2019** |
| 122 | **1/4/2019** | **250** | **Delivered** | **4/28/2019** |

| Recipient | Donors for SCNT | No. of embryos | Pregnant sows | Liveborn | Stillborn | No. of weaned piglets |
| --- | --- | --- | --- | --- | --- | --- |
| No.10 | 25#,112# (cell colonies) | 354 | Delivered | 5 | 2 | 1 (1085#) |
| No.11 | 25#,112# (cell colonies) | 363 | — |  |  |  |
| No.19 | 18# (cell colony) | 336 | — |  |  |  |
| No.20 | 18# (cell colony) | 289 | — |  |  |  |
| No.21 | 25# (cell colony) | 463 | — |  |  |  |
| No.22 | 25# (cell colony) | 465 | Delivered | 3 | 0 | 3 (1143#,1144#,1145#) |
| No.28 | 89# (cell colony) | 217 | — |  |  |  |
| No.31 | 18# (cell colony) | 336 | — |  |  |  |
| No.32 | 18# (cell colony) | 318 | — |  |  |  |
| No.35 | 18# (cell colony) | 316 | — |  |  |  |
| No.36 | 18# (cell colony) | 323 | — |  |  |  |
| No.4 124 | 1145# (ear fibroblasts) | 280 | Delivered | 1 | 0 | 1 (473#) |
| No.5 LY17402 | 1145# (ear fibroblasts) | 260 | — |  |  |  |
| No.6 18421 | 1145# (ear fibroblasts) | 260 | Delivered | 4 | 0 | 4 (474#,475#,476#,477#) |
| No.7 125 | 1145# (ear fibroblasts) | 260 | — |  |  |  |
| No.8 126 | 1145# (ear fibroblasts) | 260 | Delivered | 9 | 0 | 4 (478#,481#,482#,484#) |
| No.9 127 | 1145# (ear fibroblasts) | 260 | Delivered | 6 | 3 | 3 (486#,488#,489#) |
| No.10 18445 | 1145# (ear fibroblasts) | 230 | — |  |  |  |
| No.11 LY15110 | 1145# (ear fibroblasts) | 230 | — |  |  |  |
| No.12 LY17414 | 1145# (ear fibroblasts) | 230 | — |  |  |  |
