# Supplementary material for: CD163 and pAPN double-knockout pigs are resistant to PRRSV and TGEV and exhibit decreased susceptibility to PDCoV while maintaining normal production performance
Source: eLife. 2020 Sep 2;9:e57132. doi: 10.7554/eLife.57132 (PMC7467724; doi:10.7554/eLife.57132)
Supplement: Supplementary file 3. [file elife-57132-supp3.docx]

**Supplementary file 3. Potential off-target sites identified for *CD163* sgRNA and *pAPN* sgRNA**

| Site name | Sequence | Presence of potential off-target |
| --- | --- | --- |
| *CD163* On-target | **GGAAACCCAGGCTGGTTGGA**GGG |  |
| *CD163* OT1 | **AGAAACCCACCCTGGTTGGA**GGG | NO |
| *CD163* OT2 | **GGACACCCTAGCTGGTTGGA**TGG | NO |
| *CD163* OT3 | **AGCATCCAAGGCTGGTTGGA**GGG | NO |
| *CD163* OT4 | **GGAGACCCAGGCTGGTTGGG**CAG | NO |
| *CD163* OT5 | **GAAAACCCTGGCTGGTTGGT**TGG | NO |
| *CD163* OT6 | **GAAAAACCAGGCTGGTTGGC**GGG | NO |
| *CD163* OT7 | **GAAAACCCTGGCTGGTTGGA**AGA | NO |
| *CD163* OT8 | **GGAAACAGAGGCTGGTTGGG**TGG | NO |
| *CD163* OT9 | **TGAAGCCCAGGCTGCTTGGA**AGG | NO |
| *CD163* OT10 | **TGAGCCCCCGGCTGGTTGGA**GGG | NO |
| *pAPN* On-target | **GCATCCTCCTCGGCGTGG**CGG |  |
| *pAPN* OT1 | **GCATCCTCCTGGGTGTGG**TGG | NO |
| *pAPN* OT2 | **GTCTCCTGCTCGGCGTGG**AGG | NO |
| *pAPN* OT3 | **GGGTCCTTCTCGGCGTGG**GGG | NO |
| *pAPN* OT4 | **GACTCCTCCTCGGCTTGG**TGG | NO |
| *pAPN* OT5 | **GCATGGTGCTCGGCGTGG**GGG | NO |
| *pAPN* OT6 | **GCTCCCTTCTCGGCGTGG**AGG | NO |
| *pAPN* OT7 | **ACATCCTCTTCGGCGTGG**TGG | NO |
| *pAPN* OT8 | **GCAGCCTCCTCGGCCTGG**GGG | NO |
| *pAPN* OT9 | **GCATCACCCTGGGCGTGG**TGG | NO |
| *pAPN* OT10 | **GCGTCCTGCTGGGTGTGG**TGG | NO |
