# Supplementary material for: CD163 and pAPN double-knockout pigs are resistant to PRRSV and TGEV and exhibit decreased susceptibility to PDCoV while maintaining normal production performance
Source: eLife. 2020 Sep 2;9:e57132. doi: 10.7554/eLife.57132 (PMC7467724; doi:10.7554/eLife.57132)
Supplement: Supplementary file 4. [file elife-57132-supp4.docx]

**Supplementary file 4. Amino acid content of DKO lean meat and WT lean meat**

| Item | Mean ± SEM of WT | Mean ± SEM of DKO | p Value |  |
| --- | --- | --- | --- | --- |
| Asp (%) | 2.223 ± 0.07965 N = 3 | 2.177 ± 0.03480 N = 3 | 0.6198 | ns |
| Thr (%) | 1.053 ± 0.03383 N = 3 | 1.033 ± 0.01764 N = 3 | 0.6278 | ns |
| Ser (%) | 0.8733 ± 0.01856 N = 3 | 0.8700 ± 0.01528 N = 3 | 0.8964 | ns |
| Glu (%) | 3.737 ± 0.1367 N = 3 | 3.627 ± 0.08667 N = 3 | 0.5340 | ns |
| Gly (%) | 0.9933 ± 0.02906 N = 3 | 0.9900 ± 0.01528 N = 3 | 0.9240 | ns |
| Ala (%) | 1.347 ± 0.03283 N = 3 | 1.350 ± 0.03215 N = 3 | 0.9456 | ns |
| Cys (%) | 0.2033 ± 0.006667 N = 3 | 0.2033 ± 0.003333 N = 3 | > 0.9999 | ns |
| Val (%) | 1.200 ± 0.03215 N = 3 | 1.193 ± 0.02333 N = 3 | 0.8749 | ns |
| Met (%) | 0.6567 ± 0.02667 N = 3 | 0.6300 ± 0.01528 N = 3 | 0.4345 | ns |
| Ile (%) | 1.050 ± 0.03512 N = 3 | 1.037 ± 0.01667 N = 3 | 0.7489 | ns |
| Leu (%) | 2.013 ± 0.06839 N = 3 | 2.000 ± 0.04000 N = 3 | 0.8745 | ns |
| Tyr (%) | 0.7300 ± 0.02517 N = 3 | 0.7233 ± 0.01202 N = 3 | 0.8228 | ns |
| Phe (%) | 0.8800 ± 0.03055 N = 3 | 0.8467 ± 0.02333 N = 3 | 0.4348 | ns |
| Lys (%) | 2.107 ± 0.07219 N = 3 | 2.043 ± 0.03930 N = 3 | 0.4840 | ns |
| His (%) | 0.9867 ± 0.01764 N = 3 | 1.003 ± 0.008819 N = 3 | 0.4456 | ns |
| Arg (%) | 1.490 ± 0.05033 N = 3 | 1.440 ± 0.03000 N = 3 | 0.4416 | ns |
| Pro (%) | 0.8133 ± 0.02333 N = 3 | 0.7967 ± 0.01202 N = 3 | 0.5599 | ns |
| Trp (%) | 0.2133 ± 0.008819 N = 3 | 0.2067 ± 0.008819 N = 3 | 0.6213 | ns |

ns, p > 0.05.
