# Supplementary material for: CD163 and pAPN double-knockout pigs are resistant to PRRSV and TGEV and exhibit decreased susceptibility to PDCoV while maintaining normal production performance
Source: eLife. 2020 Sep 2;9:e57132. doi: 10.7554/eLife.57132 (PMC7467724; doi:10.7554/eLife.57132)
Supplement: Supplementary file 5. [file elife-57132-supp5.docx]

**Supplementary file 5. Primers for genotyping**

| Primer sets | Sequences (5’–3’) | Products |
| --- | --- | --- |
| *CD163-*F/*CD163-*R | AAGCCCACTGTAGGCAGAA | 300 bp or 292 bp |
|  | CCCCAGGAGGGAAACCAC |  |
| *pAPN*-F/*pAPN*-R | TACCCAGTTCAGTGACCTTCGTC | 286 bp |
|  | TGCTCGGCATTCTTGTTCTTCT |  |
