# Supplementary material for: CD163 and pAPN double-knockout pigs are resistant to PRRSV and TGEV and exhibit decreased susceptibility to PDCoV while maintaining normal production performance
Source: eLife. 2020 Sep 2;9:e57132. doi: 10.7554/eLife.57132 (PMC7467724; doi:10.7554/eLife.57132)
Supplement: Supplementary file 6. [file elife-57132-supp6.docx]

**Supplementary file 6. Primers for PCR detection of random integration**

| Primer sets | Sequences (5’–3’) | Products |
| --- | --- | --- |
| F1/R1 | GGAAAGGACGAAACACCG | 523 bp |
|  | AGTGAAGCAGAACGTGGG |  |
| F2/R2 | GGAGCACCTGCCTGAAAT | 536 bp |
|  | TCCACGATGTTGCCGAAG |  |
| F3/R3 | CAGCTGGTGCAGACCTACAA | 869 bp |
|  | CAGGGGGTGATGGTTTCCTC |  |
| F4/R4 | AGATGATCGCCAAGAGCGAG | 439 bp |
|  | ATCCCCAGCAGCTCTTTCAC |  |
| F5/R5 | CAGACAAGCTGTGACCGTCT | 525 bp |
|  | CCGGCGTCAATACGGGATAA |  |
| F6/R6 | ATCTACACGACGGGGAGTCA | 707 bp |
|  | CCGCTTACCGGATACCTGTC |  |
