# Supplementary material for: CD163 and pAPN double-knockout pigs are resistant to PRRSV and TGEV and exhibit decreased susceptibility to PDCoV while maintaining normal production performance
Source: eLife. 2020 Sep 2;9:e57132. doi: 10.7554/eLife.57132 (PMC7467724; doi:10.7554/eLife.57132)
Supplement: Supplementary file 7. [file elife-57132-supp7.docx]

**Supplementary file 7. Primers for off-target analysis**

| Primer sets | Sequences (5’–3’) | Products |
| --- | --- | --- |
| *CD163*-OT1-F/*CD163*-OT1-R | AACTGTAGCGGGAGGAGGC | 297 bp |
|  | CAAAACGGTCCGAAAGAGC |  |
| *CD163*-OT2-F/*CD163*-OT2-R | GAGTTGGTGGTTCTGGAGTTAGG | 238 bp |
|  | CCGGCCTGGAGTGGTCTC |  |
| *CD163*-OT3-F/*CD163*-OT3-R | TGGGACAGTGAGCGAAAG | 422 bp |
|  | AAGGAACAGTGGCTGGATT |  |
| *CD163*-OT4-F/*CD163*-OT4-R | GCGTTACCTCTGCTCAAA | 508 bp |
|  | CAGTGTCCAGCGACTTCA |  |
| *CD163*-OT5-F/*CD163*-OT5-R | ACTTCTAGTGGAGCAAGTAA | 350 bp |
|  | GGGTGTATGTGGGAATAA |  |
| *CD163*-OT6-F/*CD163*-OT6-R | CCTGGATAAAGATGAGGAAAC | 410 bp |
|  | CCTTAGCCTGTGAGGAGAAT |  |
| *CD163*-OT7-F/*CD163*-OT7-R | AAAGCCACCCTGTATGAA | 226 bp |
|  | GAGACAATTATGGCAGTC |  |
| *CD163*-OT8-F/*CD163*-OT8-R | TTTGGAATTTGGCCTTCT | 306 bp |
|  | AATGCCCTATCCTTAACCC |  |
| *CD163*-OT9-F/*CD163*-OT9-R | GAACTCATCTCACCCGTTAA | 379 bp |
|  | ATTGCTTCCTTCCTGTCA |  |
| *CD163*-OT10-F/*CD163*-OT10-R | GGGAGGACATGGAACTCA | 500 bp |
|  | GGCTGGAGCATTCACTCA |  |
| *pAPN*-OT1-F/*pAPN*-OT1-R | CTGGTGCTGTGCATGGGAATC | 215 bp |
|  | TGTGGGCTTGGCTGTGCTG |  |
| *pAPN*-OT2-F/*pAPN*-OT2-R | CCTCTATACGATTTCCCACAG | 255 bp |
|  | CGAAAGGTCTCGTCCACC |  |
| *pAPN*-OT3-F/*pAPN*-OT3-R | TCCTTTACAAAATCAAAGATGAC | 272 bp |
|  | CTTGCTTCTATTCCCGAGT |  |
| *pAPN*-OT4-F/*pAPN*-OT4-R | AGGTTCCCTCTTGTTCTTTC | 365 bp |
|  | ACTTCAAGGCTTCTGCTCTA |  |
| *pAPN*-OT5-F/*pAPN*-OT5-R | AAGCACCTTCTCAGTCCTCA | 550 bp |
|  | ATGCCACCTCCCTTCTCC |  |
| *pAPN*-OT6-F/*pAPN*-OT6-R | CTGTTGTCGTCCGTGGCTGAG | 266bp |
|  | TGGTCTCCCACTGGCTGCTC |  |
| *pAPN*-OT7-F/*pAPN*-OT7-R | TTCCAGATCGGGTCACATTCC | 534 bp |
|  | CATCAGGTTGAACTTGAGGGACTT |  |
| *pAPN*-OT8-F/*pAPN*-OT8-R | TTATGCCACTGCTTCAACTC | 384 bp |
|  | GACCACCTTCCCTGCTCT |  |
| *pAPN*-OT9-F/*pAPN*-OT9-R | CCCCTTGGCAGTGAGCAT | 265 bp |
|  | CTTTAGGTCCAGCAGGGTGTAG |  |
| *pAPN*-OT10-F/*pAPN*-OT10-R | CTCGGGGAGGGTTTTGAA | 234 bp |
|  | GCTGGAGTCCTGGCGTGA |  |
