# Supplementary material for: CD163 and pAPN double-knockout pigs are resistant to PRRSV and TGEV and exhibit decreased susceptibility to PDCoV while maintaining normal production performance
Source: eLife. 2020 Sep 2;9:e57132. doi: 10.7554/eLife.57132 (PMC7467724; doi:10.7554/eLife.57132)
Supplement: Supplementary file 8. [file elife-57132-supp8.docx]

**Supplementary file 8.** **Birth weights and average daily gains of of WT pigs and DKO pigs from birth weight to slaughtering weight**

| Item | Mean ± SEM of WT | Mean ± SEM of DKO | p Value |  |
| --- | --- | --- | --- | --- |
| Birth weight (kg) | 1.273 ± 0.02028 N=3 | 1.267 ± 0.06173 N=3 | 0.9232 | ns |
| Average daily gain (g) | 482.8 ± 22.27 N=3 | 491.9 ± 9.643 N=3 | 0.7265 | ns |

ns, p > 0.05.
